# Crystal structures of 2-[(4,6-di­amino­pyrimidin-2-yl)sulfan­yl]-*N*-(2,4-di­methyl­phen­yl)acetamide and 2-[(4,6-di­amino­pyrimidin-2-yl)sulfan­yl]-*N*-(3-meth­oxy­phen­yl)acetamide

**DOI:** 10.1107/S2056989017008143

**Published:** 2017-06-13

**Authors:** Manisha Choudhury, Vijayan Viswanathan, Ajay Kumar Timiri, Barij Nayan Sinha, Venkatesan Jayaprakash, Devadasan Velmurugan

**Affiliations:** a Centre of Advanced Study in Crystallography and Biophysics, University of Madras, Guindy Campus, Chennai 600 025, India; b Department of Pharmaceutical Science and Technology, Birla Institute of Technology, Mesta, Ranchi 835 215, Jharkhand, India

**Keywords:** crystal structure, di­amino­pyrimidine, pharmacological properties, hydrogen bonding

## Abstract

In the title compounds, C_14_H_17_N_5_OS (I) and C_13_H_15_N_5_O_2_S (II), the dihedral angle between the pyrimidine and benzene rings is 58.64 (8)° in (I) and 78.33 (9)° in (II). In both compounds, there is an intra­molecular C—H⋯O hydrogen bond, and in (II) there is also an intra­molecular N—H⋯N hydrogen bond present. In the crystals of both compounds, a pair of N—H⋯N hydrogen bonds links the individual mol­ecules to form inversion dimers with *R*_2_^2^(8) ring motifs. In (I), the dimers are linked by N—H⋯O and C—H⋯O hydrogen bonds, enclosing *R*_2_^1^(14), *R*_2_^1^(11) and *R*_2_^1^(7) ring motifs, forming layers parallel to the (100) plane. There is also an N—H⋯π inter­action present within the layer. In (II), the inversion dimers are linked by N—H⋯O hydrogen bonds enclosing an *R*_4_^4^(18) ring motif. The presence of N—H⋯O and C—H⋯O hydrogen bonds generate an *R*_2_^1^(6) ring motif. The combination of these various hydrogen bonds results in the formation of layers parallel to the (1-11) plane.

## Abstract 2

Two 2-[(4,6-di­amino­pyrimidin-2-yl)sulfan­yl]acetamide derivatives have folded conformations with the pyrimidine ring being inclined to the benzene ring by 58.64 (8) and 78.33 (9)°.

## Chemical context   

Di­amino­pyrimidine derivatives have been proved to be an important class of compounds because of their therapeutic and pharmacological properties. One such important property is its inhibition potency against cancer targets. As a result of the limited capacity of drugs that can cure or at least prolong the survival of cancer patients, there is always an strong requirement for new chemotherapeutics. It has been reported that di­amino­pyrimidines show inhibition against cyclin-dependent kinases (cdks), thus arresting cell proliferation in cancer cells (Mesguiche *et al.*, 2003 ▸). 2,4-Di­amino­pyrimidine derivatives have also shown effective suppression of anaplastic lymphoma kinase (ALK), one of the receptor tyrosine kinases that is involved in a variety of tumours (Achary *et al.*, 2017 ▸). 2,4-Di­amino­pyrimidine derivatives have also been reported to exhibit potent inhibitory activity against influenza viruses (Kimura *et al.*, 2006 ▸) and have anti-retroviral activity (Hocková *et al.*, 2004 ▸), anti-bacterial (Kandeel *et al.*, 1994 ▸) and potential anti-microbial properties (Holla *et al.*, 2006 ▸). Several di­amino­pyrimidine derivatives have shown good activity, efficiency against the malarial parasite *Plasmodium falciparum* K1 strain (Phuangsawai *et al.*, 2016 ▸; Chiang *et al.*, 2009 ▸). Inter­estingly, they also act as calcium channel blocking agents (Manjula *et al.*, 2004 ▸; Singh *et al.*, 2009 ▸). As part of our own studies in this area, we report herein on the syntheses and crystal structure analyses of the title compounds, 2-[(4,6-di­amino­pyrimidin-2-yl)sulfan­yl]-*N*-(2,4-di­methyl­phen­yl)acet­amide (I) and [2-((4,6-di­amino­pyrimidin-2-yl)sulfan­yl]-*N*-(3-meth­oxy­phen­yl)acetamide (II).
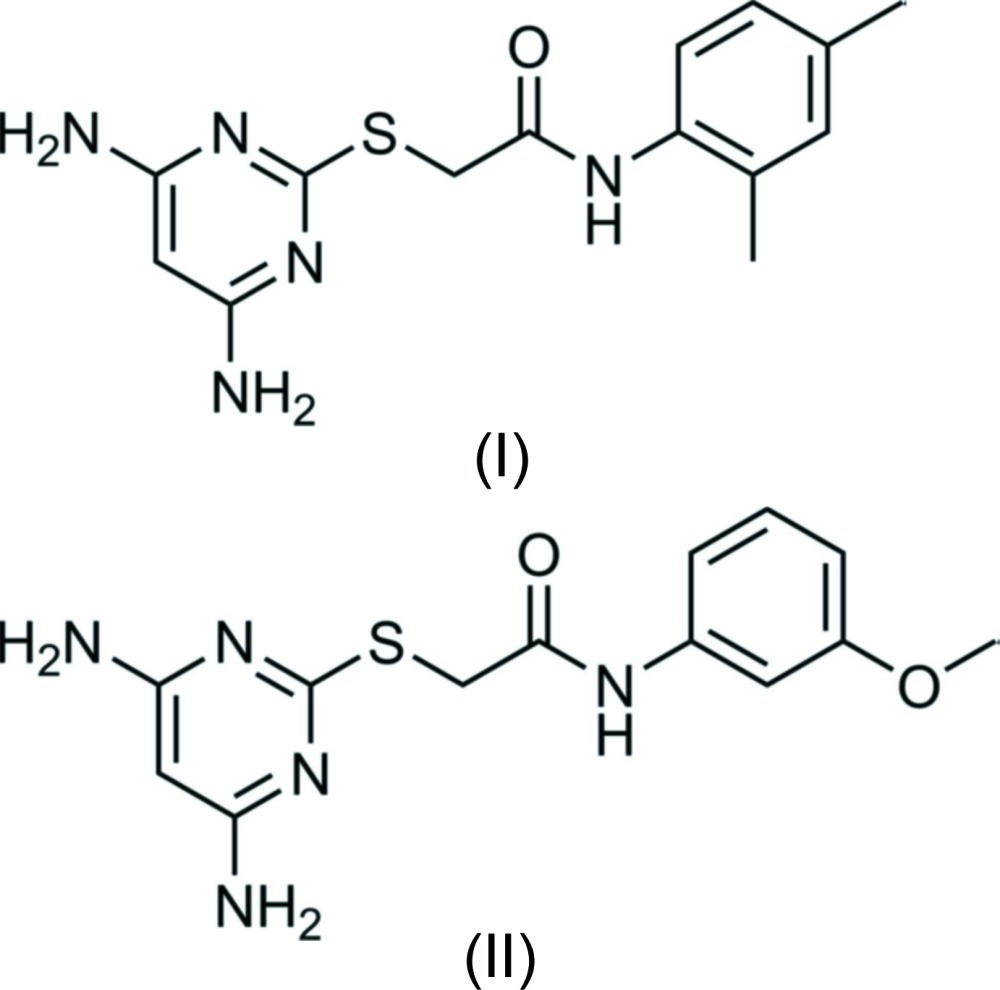


## Structural commentary   

**Figure 1:**
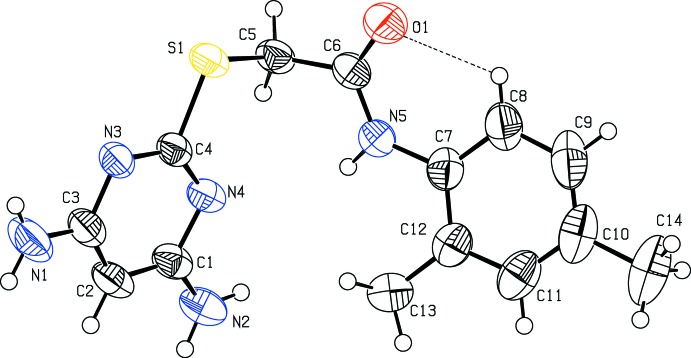
The mol­ecular structure of (I), showing the atom labelling and displacement ellipsoids drawn at 50% probability level. The C—H⋯O contact is shown as a dashed line (see Table 1 ▸).

The mol­ecular structures of compounds (I) and (II) are shown in Figs. 1 ▸ and 2 ▸, respectively. Compound (I) crystallizes in the monoclinic space group *P*2_1_/*c* and compound (II) in the triclinic space group *P* 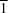. In compounds (I) and (II), the di­amino­pyrimidine and benzene rings are inclined to one another by 58.64 (8) and 78.33 (9)°, respectively. The torsion angle C4—S1—C5—C6 = 98.12 (11) ° in compound (I) and −80.14 (14) ° in compound (II), torsion angles S1—C5—C6—N5 = −101.92 (14) ° in compound (I) and 82.23 (16) ° in compound (II), and C5—C6—N5—C7 = 178.66 (15)° in compound (I) and −172.71 (14) ° in compound (II). The bond lengths C4—S1 = 1.7650 (14) Å and C5—S1 = 1.8053 (16) Å in compound (I), and C4—S1 = 1.7721 (17) Å and C5—S1 = 1.8126 (18) Å in compound (II), are comparable with the values reported for a similar structure, 2-[(4,6-di­amino­pyrimidin-2-yl)sulfan­yl]-*N*-(2-methyl­phen­yl)acetamide, *viz.* 1.763 and 1.805 Å, respectively (Subasri *et al.*, 2014 ▸). In compound (I), atoms C13 and C14 deviate from the benzene ring by 0.010 (3) and 0.012 (3) Å, respectively. Atoms N1 and N2 deviate from the mean plane of the pyrimidine ring by −0.0819 (18) and 0.0636 (14) Å, respectively, in compound (I), and by 0.0360 (3) and 0.0273 (3) Å, respectively, in compound (II). In both compounds, an intra­molecular hydrogen bond, C8—H8⋯O1, forms an *S*(6) ring motif, and in compound (II) there is also an intra­molecular N—H⋯N hydrogen bond present that forms an *S*(7) ring motif (see Tables 1 ▸ and 2 ▸).

## Supra­molecular features   

**Figure 3:**
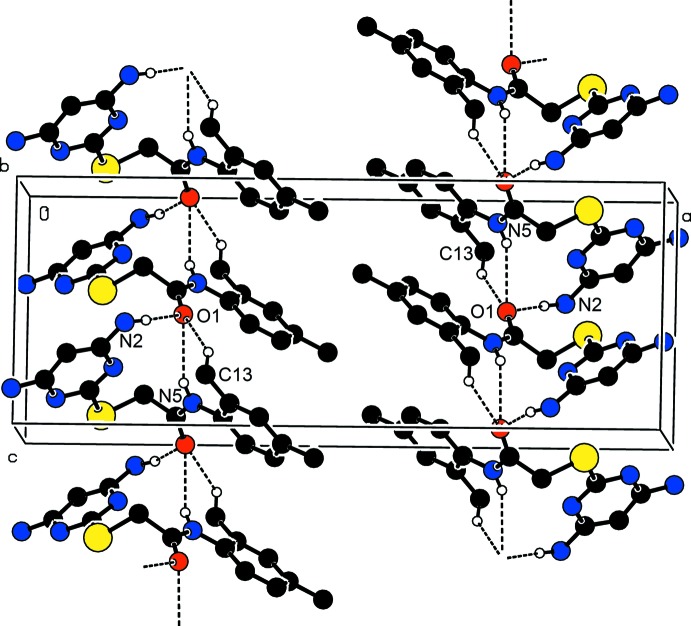
The crystal packing of (I), viewed along the *b* axis, C—H⋯O and N—H⋯O hydrogen bonds generate 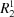
(14), 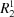
(11) and 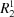
(7) ring motifs. In this and subsequent figures, the hydrogen bonds are shown as dashed lines and H atoms not involved in hydrogen bonding have been omitted for clarity.

**Figure 4:**
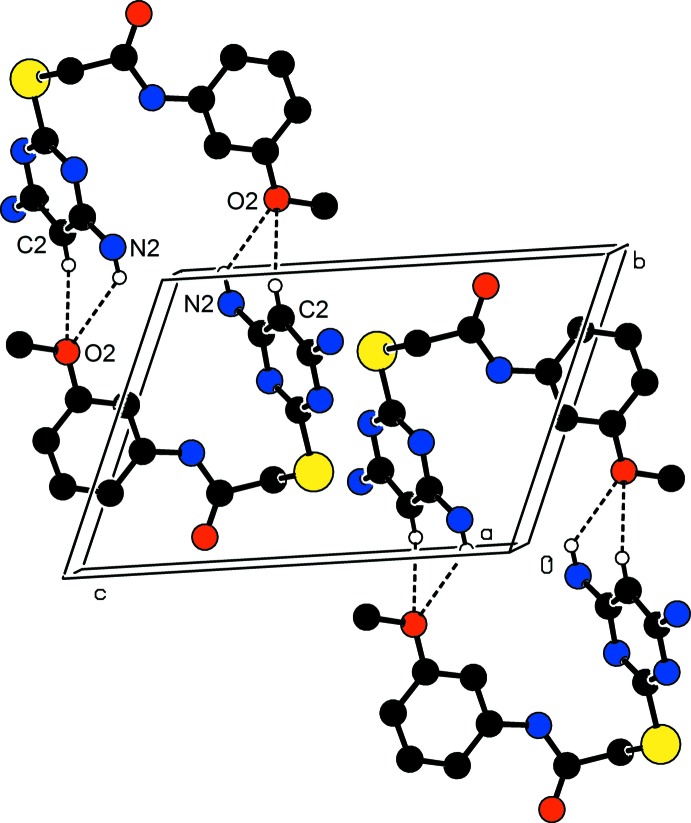
A partial view of the crystal packing of (I), viewed approximately along the *c* axis, showing the N—H⋯π inter­actions.

**Figure 5:**
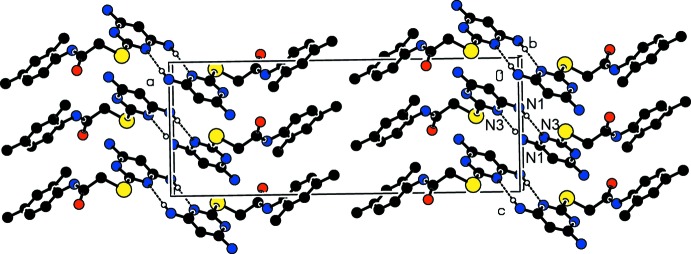
A view along the *b* axis of the crystal packing of (I), showing the N—H⋯N hydrogen bonds that generate an 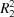
(8) ring motif.

The hydrogen-bonding geometry of compounds (I) and (II) are given in Tables 1 ▸ and 2 ▸, respectively. In compound (I), atom O1 is a triple acceptor of hydrogen bonds. The N5—H5⋯O1^ii^ hydrogen bonds form a chain running along the *c-*axis direction. The N2—H2*A*⋯O1^ii^ and C13—H13*C*⋯O1^ii^ hydrogen bonds generate an 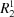
(14) ring motif, and the N2—H2*A*⋯O1^ii^ and N5—H5⋯O1^ii^ hydrogen bonds form an 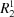
(11) ring motif, and N5—H5⋯O1^ii^ and C13—H13⋯O1^ii^ hydrogen bonds generate an 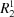
(7) ring motif (Table 1 ▸ and Fig. 3 ▸). There is also a N2—H2*B*⋯π inter­action present within the layer (Table 1 ▸ and Fig. 4 ▸), with the separation distance between the donor and acceptor, *Cg*1, being 3.4851 (1) Å. The N1—H1*A*⋯N3^i^ hydrogen bond generates an inversion dimer with an 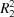
(8) ring motif (Table 1 ▸ and Fig. 5 ▸). As a result of the hydrogen bonding, layers parallel to the *bc* plane are formed.

**Figure 6:**
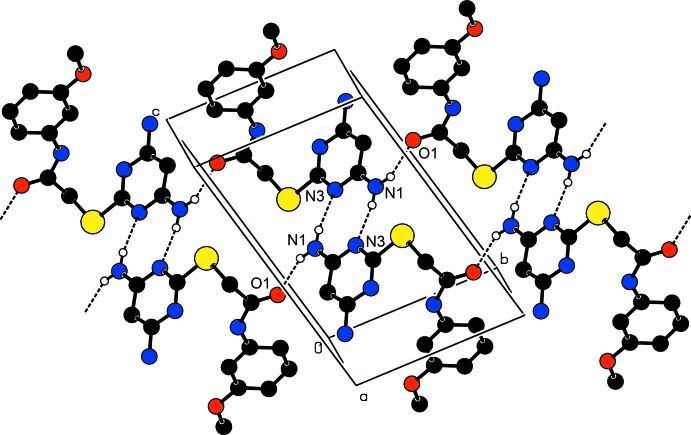
The crystal packing of (II), viewed along the *a* axis, C—H⋯O and N—H⋯O hydrogen bonds generate two 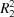
(26) ring motifs and one 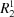
(6) ring motif.

**Figure 7:**
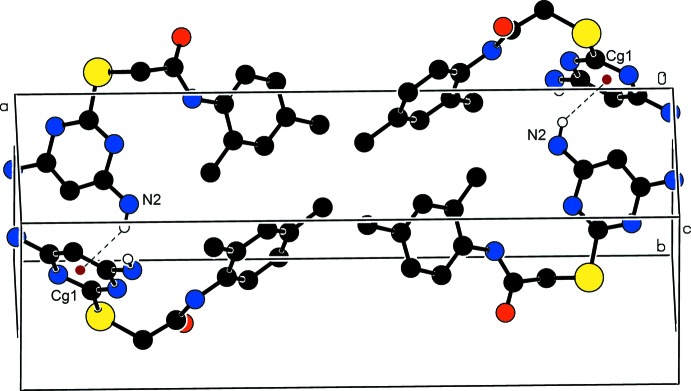
A partial view along the *a* axis of the crystal packing of (II). The N—H⋯N hydrogen bonds generate an 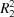
(8) ring motif and N—H⋯O and N—H⋯N hydrogen bonds an 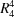
(18) ring motif.

In compound (II), atom O2 is a double acceptor of hydrogen bonds. The N2—H2*B*⋯O2^iii^ hydrogen bond forms an 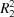
(26) ring motif and hydrogen bond C2—H2⋯O2^iii^ generates an 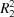
(26) ring motif (Table 2 ▸ and Fig. 6 ▸). These two inter­molecular hydrogen bonds generate an 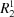
(6) ring motif, which is shown in Fig. 6 ▸. Mol­ecules are linked by a pair of N1—H1*A*⋯N3^i^ hydrogen bonds, forming an inversion dimer with an 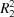
(8) ring motif, and hydrogen bonds N1—H1*B*⋯O1^ii^ and N1—H1*A*⋯N3^i^ generate an 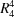
(18) ring motif (Table 2 ▸ and Fig. 7 ▸). The combination of these various hydrogen bonds results in the formation of layers parallel to (1
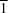
1).

## Database survey   

A search of the Cambridge Structure Database (Version 5.37, update May 2016; Groom *et al.*, 2016 ▸) for 2-[(pyrimidine-2-yl)sulfan­yl]-*N*-phenyl­acetamide yielded seven hits. Three of these involve (4,6-di­amino­pyrmidin-2-yl) groups. They include the 2-methyl­phenyl analogue, 2-[(4,6-di­amino­pyrimidin-2-yl)sulfan­yl]-*N*-(2-methyl­phen­yl)acetamide (GOKWIO; Subasri *et al.*, 2014 ▸), the 2-chloro­phenyl analogue, *N*-(2-chloro­phen­yl)-2-[(4,6-di­amino­pyrimidin-2-yl) sulfan­yl]acetamide (ARARUI; Subasri *et al.*, 2016 ▸) and the 3-nitro­phenyl analogue, 2-[(4,6-di­amino­pyrimidin-2-yl)sulfan­yl]-*N*-(3-nitro­phen­yl)acetamide (ARAROC; Subasri *et al.*, 2016 ▸). Here the pyrimidine and benzene rings are inclined to one another by 54.73, 67.11 and 56.19°, respectively, compared to 58.64 (8) ° in compound (I), and 78.33 (9) ° in compound (II).

## Synthesis and crystallization   

**Compound (I)**: To a solution of 4,6-di­amino-pyrimidine-2-thiol (0.5 g, 3.52 mmol) in 25 ml of ethanol in a round-bottom flask, potassium hydroxide (0.2 g, 3.52 mmol) was added and the mixture was refluxed for 30 min. 2,4-Di­methyl­phenyl acetamide (3.52 mmol) was added and the mixture was refluxed for 3 h. At the end of the reaction (observed by TLC), the ethanol was evaporated under vacuum and cold water was added. The precipitate formed was filtered and dried to give compound (I) as a crystalline powder (yield 67%). After purification, the compound was recrystallized from ethanol solution by slow evaporation of the solvent.

**Compound (II)**: To a solution of 4,6-di­amino-pyrimidine-2-thiol (0.5 g, 3.52 mmol) in 25 ml of ethanol in a round-bottom flask was added potassium hydroxide (0.2 g, 3.52 mmol) and the mixture was refluxed for 30 min. 3-Meth­oxy­phenyl acetamide (3.52 mmol) was added and the mixture was refluxed for 3 h. At the end of the reaction (observed by TLC), the ethanol was evaporated under vacuum and cold water was added, and the precipitate formed was filtered and dried to give compound (II) as a shiny powder (yield 73%). After purification, the compound was recrystallized from ethanol solution by slow evaporation of the solvent.

## Refinement   

Crystal data, data collection and structure refinement details are summarized in Table 3 ▸. For both compounds the hydrogen atoms were placed in calculated positions and refined using the riding model: C—H = 0.93–0.97 Å and N—H = 0.86 Å, with *U*_iso_(H) = 1.5*U*_eq_(C-meth­yl) and 1.2*U*_eq_(N,C) for other H atoms.

## Supplementary Material

Crystal structure: contains datablock(s) global, I, II. DOI: 10.1107/S2056989017008143/su5373sup1.cif

Structure factors: contains datablock(s) I. DOI: 10.1107/S2056989017008143/su5373Isup2.hkl

Structure factors: contains datablock(s) II. DOI: 10.1107/S2056989017008143/su5373IIsup3.hkl

Supporting information file. DOI: 10.1107/S2056989017008143/su5373Isup4.cml

Supporting information file. DOI: 10.1107/S2056989017008143/su5373IIsup5.cml

CCDC references: 1553497, 1553496

Additional supporting information:  crystallographic information; 3D view; checkCIF report

## Figures and Tables

**Figure 2:**
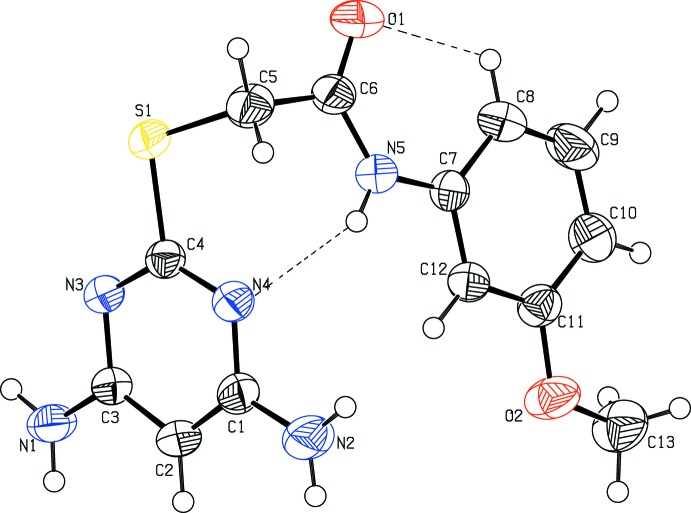
The mol­ecular structure of (II), showing the atom labelling and displacement ellipsoids drawn at 50% probability level. The N—H⋯N and C—H⋯O contacts are shown as dashed lines (see Table 2 ▸).

**Table 1:** Hydrogen-bond geometry (Å, °) for (I) *Cg*1 is the centroid of the N3/N4/C1–C4 ring.

*D*—H⋯*A*	*D*—H	H⋯*A*	*D*⋯*A*	*D*—H⋯*A*
C8—H8⋯O1	0.93	2.30	2.8752 (1)	120
N1—H1*A*⋯N3^i^	0.86	2.26	3.1187 (1)	175
N2—H2*A*⋯O1^ii^	0.86	2.32	3.1032 (1)	152
N5—H5⋯O1^ii^	0.86	2.51	3.2640 (1)	146
C13—H13*C*⋯O1^ii^	0.96	2.56	3.3880 (1)	144
N2—H2*B*⋯*Cg*1^iii^	0.86	2.88	3.4851 (1)	130

Symmetry codes: (i) 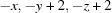; (ii) 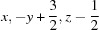; (iii) 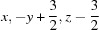.

**Table 2:** Hydrogen-bond geometry (Å, °) for (II)

*D*—H⋯*A*	*D*—H	H⋯*A*	*D*⋯*A*	*D*—H⋯*A*
N5—H5⋯N4	0.86	2.15	2.861 (3)	140
C8—H8⋯O1	0.93	2.34	2.911 (3)	120
N1—H1*A*⋯N3^i^	0.86	2.21	3.035 (3)	162
N1—H1*B*⋯O1^ii^	0.86	2.08	2.891 (3)	157
N2—H2*B*⋯O2^iii^	0.86	2.55	3.210 (3)	135
C2—H2⋯O2^iii^	0.93	2.59	3.272 (3)	130

Symmetry codes: (i) 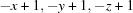; (ii) 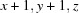; (iii) 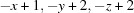.

**Table 3:** Experimental details

	(I)	(II)
Crystal data		
Chemical formula	C_14_H_17_N_5_OS	C_13_H_15_N_5_O_2_S
*M*_r_	303.38	305.36
Crystal system, space group	Monoclinic, *P*2_1_/*c*	Triclinic, *P* 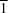
Temperature (K)	293	293
*a*, *b*, *c* (Å)	23.7716 (6), 7.0073 (2), 9.0909 (2)	8.014 (5), 8.724 (5), 12.068 (5)
α, β, γ (°)	90, 90.086 (2), 90	106.561 (5), 97.888 (5), 110.461 (5)
*V* (Å^3^)	1514.31 (7)	730.9 (7)
*Z*	4	2
Radiation type	Mo *K*α	Mo *K*α
μ (mm^−1^)	0.22	0.23
Crystal size (mm)	0.24 × 0.18 × 0.12	0.30 × 0.25 × 0.20

Data collection		
Diffractometer	Bruker SMART APEXII area-detector	Bruker SMART APEXII area-detector
Absorption correction	Multi-scan (*SADABS*; Bruker, 2008 ▸)	Multi-scan (*SADABS*; Bruker, 2008 ▸)
*T*_min_, *T*_max_	0.785, 0.854	0.785, 0.843
No. of measured, independent and observed [*I* > 2σ(*I*)] reflections	13879, 3705, 2750	10789, 2991, 2616
*R*_int_	0.022	0.026
(sin θ/λ)_max_ (Å^−1^)	0.666	0.627

Refinement		
*R*[*F* ^2^ > 2σ(*F* ^2^)], *wR*(*F* ^2^), *S*	0.040, 0.119, 1.06	0.037, 0.110, 0.81
No. of reflections	3705	2991
No. of parameters	192	191
H-atom treatment	H-atom parameters constrained	H-atom parameters constrained
Δρ_max_, Δρ_min_ (e Å^−3^)	0.22, −0.24	0.17, −0.27

Computer programs: *APEX2* and *SAINT* (Bruker, 2008 ▸), *SHELXS97* (Sheldrick, 2008 ▸), *SHELXL2016* (Sheldrick, 2015 ▸), *ORTEP-3 for Windows* (Farrugia, 2012 ▸) and *PLATON* (Spek, 2009 ▸).

## supplementary crystallographic information

### (I) 2-[(4,6-Diaminopyrimidin-2-yl)sulfanyl]-N-(2,4-dimethylphenyl)acetamide Crystal data

**Table:**

C_14_H_17_N_5_OS	*F*(000) = 640
*M**_r_* = 303.38	*D*_x_ = 1.331 Mg m^−^^3^
Monoclinic, *P*2_1_/*c*	Mo *K*α radiation, λ = 0.71073 Å
*a* = 23.7716 (6) Å	Cell parameters from 3705 reflections
*b* = 7.0073 (2) Å	θ = 1.7–28.3°
*c* = 9.0909 (2) Å	µ = 0.22 mm^−^^1^
β = 90.086 (2)°	*T* = 293 K
*V* = 1514.31 (7) Å^3^	Block, yellow
*Z* = 4	0.24 × 0.18 × 0.12 mm

### (I) 2-[(4,6-Diaminopyrimidin-2-yl)sulfanyl]-N-(2,4-dimethylphenyl)acetamide Data collection

**Table:**

Bruker SMART APEXII area-detector diffractometer	2750 reflections with *I* > 2σ(*I*)
ω and φ scans	*R*_int_ = 0.022
Absorption correction: multi-scan (*SADABS*; Bruker, 2008)	θ_max_ = 28.3°, θ_min_ = 1.7°
*T*_min_ = 0.785, *T*_max_ = 0.854	*h* = −31→31
13879 measured reflections	*k* = −8→9
3705 independent reflections	*l* = −12→11

### (I) 2-[(4,6-Diaminopyrimidin-2-yl)sulfanyl]-N-(2,4-dimethylphenyl)acetamide Refinement

**Table:**

Refinement on *F*^2^	Primary atom site location: structure-invariant direct methods
Least-squares matrix: full	Secondary atom site location: difference Fourier map
*R*[*F*^2^ > 2σ(*F*^2^)] = 0.040	Hydrogen site location: inferred from neighbouring sites
*wR*(*F*^2^) = 0.119	H-atom parameters constrained
*S* = 1.06	*w* = 1/[σ^2^(*F*_o_^2^) + (0.0584*P*)^2^ + 0.2187*P*] where *P* = (*F*_o_^2^ + 2*F*_c_^2^)/3
3705 reflections	(Δ/σ)_max_ < 0.001
192 parameters	Δρ_max_ = 0.22 e Å^−^^3^
0 restraints	Δρ_min_ = −0.24 e Å^−^^3^

### (I) 2-[(4,6-Diaminopyrimidin-2-yl)sulfanyl]-N-(2,4-dimethylphenyl)acetamide Special details

**Table:**

Geometry. All esds (except the esd in the dihedral angle between two l.s. planes) are estimated using the full covariance matrix. The cell esds are taken into account individually in the estimation of esds in distances, angles and torsion angles; correlations between esds in cell parameters are only used when they are defined by crystal symmetry. An approximate (isotropic) treatment of cell esds is used for estimating esds involving l.s. planes.

### (I) 2-[(4,6-Diaminopyrimidin-2-yl)sulfanyl]-N-(2,4-dimethylphenyl)acetamide Fractional atomic coordinates and isotropic or equivalent isotropic displacement parameters (Å^2^)

**Table:**

	*x*	*y*	*z*	*U*_iso_*/*U*_eq_	
S1	0.13091 (2)	0.71159 (6)	0.94117 (5)	0.05991 (16)	
N4	0.15430 (5)	1.00969 (16)	0.76929 (12)	0.0459 (3)	
N3	0.06610 (5)	0.99914 (17)	0.89584 (13)	0.0492 (3)	
C4	0.11650 (6)	0.9332 (2)	0.85688 (15)	0.0434 (3)	
O1	0.25886 (5)	0.58619 (18)	1.04523 (13)	0.0688 (4)	
C6	0.24589 (6)	0.6857 (2)	0.94031 (15)	0.0464 (3)	
N2	0.17546 (7)	1.2451 (2)	0.60612 (15)	0.0646 (4)	
H2A	0.206833	1.186878	0.592034	0.078*	
H2B	0.168182	1.348669	0.559012	0.078*	
N5	0.27421 (5)	0.84155 (19)	0.89770 (15)	0.0563 (3)	
H5	0.261074	0.898941	0.821415	0.068*	
C1	0.13761 (7)	1.1740 (2)	0.70222 (15)	0.0492 (4)	
C3	0.05202 (7)	1.1694 (2)	0.83485 (18)	0.0547 (4)	
C2	0.08687 (8)	1.2589 (2)	0.73351 (18)	0.0577 (4)	
H2	0.076240	1.372376	0.688329	0.069*	
C13	0.30123 (8)	1.2195 (2)	0.8120 (2)	0.0647 (5)	
H13A	0.314436	1.348741	0.806118	0.097*	
H13B	0.262823	1.218807	0.844537	0.097*	
H13C	0.303624	1.160894	0.716785	0.097*	
C5	0.19468 (6)	0.6392 (2)	0.84913 (17)	0.0503 (4)	
H5A	0.193461	0.502920	0.830692	0.060*	
H5B	0.197259	0.703824	0.755023	0.060*	
C12	0.33675 (7)	1.1105 (3)	0.91894 (17)	0.0562 (4)	
C7	0.32294 (7)	0.9247 (3)	0.96134 (18)	0.0565 (4)	
N1	0.00214 (7)	1.2404 (2)	0.8769 (2)	0.0826 (5)	
H1A	−0.018358	1.178344	0.938385	0.099*	
H1B	−0.009323	1.347810	0.842366	0.099*	
C11	0.38453 (8)	1.1920 (3)	0.9800 (2)	0.0705 (5)	
H11	0.394129	1.315535	0.952417	0.085*	
C8	0.35657 (8)	0.8271 (3)	1.0617 (2)	0.0766 (6)	
H8	0.347527	0.703240	1.089668	0.092*	
C10	0.41862 (8)	1.0979 (4)	1.0800 (2)	0.0808 (6)	
C9	0.40379 (8)	0.9168 (4)	1.1195 (3)	0.0893 (7)	
H9	0.425969	0.851470	1.187178	0.107*	
C14	0.47024 (9)	1.1953 (5)	1.1442 (3)	0.1146 (10)	
H14A	0.475144	1.156141	1.244577	0.172*	
H14B	0.465342	1.331180	1.140249	0.172*	
H14C	0.502847	1.160063	1.088224	0.172*	

### (I) 2-[(4,6-Diaminopyrimidin-2-yl)sulfanyl]-N-(2,4-dimethylphenyl)acetamide Atomic displacement parameters (Å^2^)

**Table:**

	*U*^11^	*U*^22^	*U*^33^	*U*^12^	*U*^13^	*U*^23^
S1	0.0529 (3)	0.0458 (2)	0.0810 (3)	0.00850 (17)	0.00160 (19)	0.01998 (19)
N4	0.0599 (8)	0.0368 (6)	0.0410 (6)	0.0054 (5)	−0.0075 (5)	−0.0003 (5)
N3	0.0506 (7)	0.0421 (7)	0.0548 (7)	0.0071 (5)	−0.0109 (5)	0.0004 (5)
C4	0.0536 (8)	0.0356 (7)	0.0410 (7)	0.0034 (6)	−0.0147 (6)	−0.0033 (5)
O1	0.0774 (8)	0.0705 (8)	0.0585 (7)	−0.0070 (6)	−0.0125 (6)	0.0206 (6)
C6	0.0516 (8)	0.0440 (8)	0.0435 (7)	0.0096 (6)	0.0034 (6)	−0.0017 (6)
N2	0.0844 (11)	0.0560 (8)	0.0535 (8)	0.0111 (7)	−0.0005 (7)	0.0154 (6)
N5	0.0545 (8)	0.0549 (8)	0.0594 (8)	−0.0026 (6)	−0.0117 (6)	0.0135 (6)
C1	0.0680 (10)	0.0419 (8)	0.0376 (7)	0.0041 (7)	−0.0122 (6)	−0.0001 (6)
C3	0.0606 (10)	0.0471 (9)	0.0565 (9)	0.0121 (7)	−0.0149 (7)	0.0009 (7)
C2	0.0735 (11)	0.0445 (8)	0.0549 (9)	0.0145 (7)	−0.0132 (8)	0.0076 (7)
C13	0.0729 (12)	0.0532 (10)	0.0679 (11)	−0.0045 (8)	0.0029 (9)	0.0017 (8)
C5	0.0584 (9)	0.0348 (7)	0.0577 (9)	0.0078 (6)	−0.0064 (7)	−0.0034 (6)
C12	0.0504 (9)	0.0651 (11)	0.0530 (9)	−0.0023 (7)	0.0089 (7)	−0.0082 (7)
C7	0.0469 (8)	0.0662 (11)	0.0564 (9)	0.0006 (7)	−0.0009 (7)	0.0014 (8)
N1	0.0686 (10)	0.0731 (11)	0.1062 (13)	0.0325 (8)	0.0045 (9)	0.0260 (9)
C11	0.0574 (10)	0.0841 (14)	0.0700 (11)	−0.0136 (9)	0.0119 (9)	−0.0166 (10)
C8	0.0549 (11)	0.0888 (15)	0.0860 (13)	0.0017 (10)	−0.0144 (9)	0.0159 (11)
C10	0.0484 (10)	0.1182 (19)	0.0758 (13)	−0.0087 (11)	0.0010 (9)	−0.0205 (12)
C9	0.0524 (11)	0.127 (2)	0.0887 (15)	0.0051 (12)	−0.0168 (10)	0.0071 (14)
C14	0.0601 (13)	0.167 (3)	0.117 (2)	−0.0246 (15)	−0.0090 (12)	−0.0338 (19)

### (I) 2-[(4,6-Diaminopyrimidin-2-yl)sulfanyl]-N-(2,4-dimethylphenyl)acetamide Geometric parameters (Å, º)

**Table:**

S1—C4	1.7650 (14)	C13—H13B	0.9600
S1—C5	1.8053 (16)	C13—H13C	0.9600
N4—C4	1.3157 (19)	C5—H5A	0.9700
N4—C1	1.3616 (18)	C5—H5B	0.9700
N3—C4	1.3325 (19)	C12—C11	1.386 (2)
N3—C3	1.3577 (19)	C12—C7	1.397 (3)
O1—C6	1.2209 (17)	C7—C8	1.391 (2)
C6—N5	1.340 (2)	N1—H1A	0.8600
C6—C5	1.507 (2)	N1—H1B	0.8600
N2—C1	1.350 (2)	C11—C10	1.384 (3)
N2—H2A	0.8600	C11—H11	0.9300
N2—H2B	0.8600	C8—C9	1.389 (3)
N5—C7	1.419 (2)	C8—H8	0.9300
N5—H5	0.8600	C10—C9	1.365 (3)
C1—C2	1.375 (2)	C10—C14	1.520 (3)
C3—N1	1.342 (2)	C9—H9	0.9300
C3—C2	1.389 (2)	C14—H14A	0.9600
C2—H2	0.9300	C14—H14B	0.9600
C13—C12	1.496 (2)	C14—H14C	0.9600
C13—H13A	0.9600		
			
C4—S1—C5	102.04 (7)	S1—C5—H5A	109.4
C4—N4—C1	114.62 (13)	C6—C5—H5B	109.4
C4—N3—C3	114.68 (14)	S1—C5—H5B	109.4
N4—C4—N3	129.41 (13)	H5A—C5—H5B	108.0
N4—C4—S1	119.27 (11)	C11—C12—C7	117.76 (17)
N3—C4—S1	111.33 (11)	C11—C12—C13	120.75 (17)
O1—C6—N5	124.39 (14)	C7—C12—C13	121.48 (15)
O1—C6—C5	120.58 (14)	C8—C7—C12	120.26 (16)
N5—C6—C5	115.02 (13)	C8—C7—N5	122.22 (17)
C1—N2—H2A	120.0	C12—C7—N5	117.53 (14)
C1—N2—H2B	120.0	C3—N1—H1A	120.0
H2A—N2—H2B	120.0	C3—N1—H1B	120.0
C6—N5—C7	128.82 (13)	H1A—N1—H1B	120.0
C6—N5—H5	115.6	C10—C11—C12	123.1 (2)
C7—N5—H5	115.6	C10—C11—H11	118.5
N2—C1—N4	114.07 (14)	C12—C11—H11	118.5
N2—C1—C2	124.03 (14)	C9—C8—C7	119.3 (2)
N4—C1—C2	121.90 (15)	C9—C8—H8	120.4
N1—C3—N3	115.25 (16)	C7—C8—H8	120.4
N1—C3—C2	123.37 (15)	C9—C10—C11	117.66 (18)
N3—C3—C2	121.35 (15)	C9—C10—C14	121.7 (2)
C1—C2—C3	117.79 (14)	C11—C10—C14	120.7 (2)
C1—C2—H2	121.1	C10—C9—C8	122.0 (2)
C3—C2—H2	121.1	C10—C9—H9	119.0
C12—C13—H13A	109.5	C8—C9—H9	119.0
C12—C13—H13B	109.5	C10—C14—H14A	109.5
H13A—C13—H13B	109.5	C10—C14—H14B	109.5
C12—C13—H13C	109.5	H14A—C14—H14B	109.5
H13A—C13—H13C	109.5	C10—C14—H14C	109.5
H13B—C13—H13C	109.5	H14A—C14—H14C	109.5
C6—C5—S1	111.24 (10)	H14B—C14—H14C	109.5
C6—C5—H5A	109.4		
			
C1—N4—C4—N3	−4.6 (2)	N5—C6—C5—S1	−101.92 (14)
C1—N4—C4—S1	175.79 (10)	C4—S1—C5—C6	98.12 (11)
C3—N3—C4—N4	0.5 (2)	C11—C12—C7—C8	0.0 (3)
C3—N3—C4—S1	−179.87 (10)	C13—C12—C7—C8	−179.62 (17)
C5—S1—C4—N4	−8.96 (12)	C11—C12—C7—N5	−179.55 (15)
C5—S1—C4—N3	171.36 (10)	C13—C12—C7—N5	0.8 (2)
O1—C6—N5—C7	−0.7 (3)	C6—N5—C7—C8	15.4 (3)
C5—C6—N5—C7	178.66 (15)	C6—N5—C7—C12	−165.04 (15)
C4—N4—C1—N2	−176.34 (12)	C7—C12—C11—C10	−0.1 (3)
C4—N4—C1—C2	4.7 (2)	C13—C12—C11—C10	179.58 (17)
C4—N3—C3—N1	−177.94 (14)	C12—C7—C8—C9	0.3 (3)
C4—N3—C3—C2	3.6 (2)	N5—C7—C8—C9	179.83 (18)
N2—C1—C2—C3	179.97 (15)	C12—C11—C10—C9	−0.2 (3)
N4—C1—C2—C3	−1.2 (2)	C12—C11—C10—C14	−179.48 (19)
N1—C3—C2—C1	178.45 (16)	C11—C10—C9—C8	0.5 (3)
N3—C3—C2—C1	−3.2 (2)	C14—C10—C9—C8	179.8 (2)
O1—C6—C5—S1	77.43 (16)	C7—C8—C9—C10	−0.6 (4)

### (I) 2-[(4,6-Diaminopyrimidin-2-yl)sulfanyl]-N-(2,4-dimethylphenyl)acetamide Hydrogen-bond geometry (Å, º)

Cg1 is the centroid of the N3/N4/C1–C4 ring.

**Table:**

*D*—H···*A*	*D*—H	H···*A*	*D*···*A*	*D*—H···*A*
C8—H8···O1	0.93	2.30	2.8752 (1)	120
N1—H1*A*···N3^i^	0.86	2.26	3.1187 (1)	175
N2—H2*A*···O1^ii^	0.86	2.32	3.1032 (1)	152
N5—H5···O1^ii^	0.86	2.51	3.2640 (1)	146
C13—H13*C*···O1^ii^	0.96	2.56	3.3880 (1)	144
N2—H2*B*···*Cg*1^iii^	0.86	2.88	3.4851 (1)	130

Symmetry codes: (i) −*x*, −*y*+2, −*z*+2; (ii) *x*, −*y*+3/2, *z*−1/2; (iii) *x*, −*y*+3/2, *z*−3/2.

### (II) 2-[(4,6-Diaminopyrimidin-2-yl)sulfanyl]-N-(3-methoxyphenyl)acetamide Crystal data

**Table:**

C_13_H_15_N_5_O_2_S	*Z* = 2
*M**_r_* = 305.36	*F*(000) = 320
Triclinic, *P*1	*D*_x_ = 1.388 Mg m^−^^3^
*a* = 8.014 (5) Å	Mo *K*α radiation, λ = 0.71073 Å
*b* = 8.724 (5) Å	Cell parameters from 2991 reflections
*c* = 12.068 (5) Å	θ = 1.8–26.5°
α = 106.561 (5)°	µ = 0.23 mm^−^^1^
β = 97.888 (5)°	*T* = 293 K
γ = 110.461 (5)°	Block, yellow
*V* = 730.9 (7) Å^3^	0.30 × 0.25 × 0.20 mm

### (II) 2-[(4,6-Diaminopyrimidin-2-yl)sulfanyl]-N-(3-methoxyphenyl)acetamide Data collection

**Table:**

Bruker SMART APEXII area-detector diffractometer	2616 reflections with *I* > 2σ(*I*)
ω and φ scans	*R*_int_ = 0.026
Absorption correction: multi-scan (*SADABS*; Bruker, 2008)	θ_max_ = 26.5°, θ_min_ = 1.8°
*T*_min_ = 0.785, *T*_max_ = 0.843	*h* = −10→9
10789 measured reflections	*k* = −10→10
2991 independent reflections	*l* = −15→15

### (II) 2-[(4,6-Diaminopyrimidin-2-yl)sulfanyl]-N-(3-methoxyphenyl)acetamide Refinement

**Table:**

Refinement on *F*^2^	Primary atom site location: structure-invariant direct methods
Least-squares matrix: full	Secondary atom site location: difference Fourier map
*R*[*F*^2^ > 2σ(*F*^2^)] = 0.037	Hydrogen site location: inferred from neighbouring sites
*wR*(*F*^2^) = 0.110	H-atom parameters constrained
*S* = 0.81	*w* = 1/[σ^2^(*F*_o_^2^) + (0.082*P*)^2^ + 0.3261*P*] where *P* = (*F*_o_^2^ + 2*F*_c_^2^)/3
2991 reflections	(Δ/σ)_max_ = 0.001
191 parameters	Δρ_max_ = 0.17 e Å^−^^3^
0 restraints	Δρ_min_ = −0.27 e Å^−^^3^

### (II) 2-[(4,6-Diaminopyrimidin-2-yl)sulfanyl]-N-(3-methoxyphenyl)acetamide Special details

**Table:**

Geometry. All esds (except the esd in the dihedral angle between two l.s. planes) are estimated using the full covariance matrix. The cell esds are taken into account individually in the estimation of esds in distances, angles and torsion angles; correlations between esds in cell parameters are only used when they are defined by crystal symmetry. An approximate (isotropic) treatment of cell esds is used for estimating esds involving l.s. planes.

### (II) 2-[(4,6-Diaminopyrimidin-2-yl)sulfanyl]-N-(3-methoxyphenyl)acetamide Fractional atomic coordinates and isotropic or equivalent isotropic displacement parameters (Å^2^)

**Table:**

	*x*	*y*	*z*	*U*_iso_*/*U*_eq_	
C1	0.2721 (2)	0.7765 (2)	0.75950 (14)	0.0415 (4)	
C2	0.4285 (2)	0.8303 (2)	0.72068 (14)	0.0416 (4)	
H2	0.517868	0.944735	0.755617	0.050*	
C3	0.4481 (2)	0.70658 (19)	0.62691 (13)	0.0371 (3)	
C4	0.1714 (2)	0.50538 (19)	0.61966 (13)	0.0345 (3)	
C5	−0.1544 (2)	0.2625 (2)	0.63675 (15)	0.0478 (4)	
H5A	−0.175956	0.368379	0.661775	0.057*	
H5B	−0.271283	0.165803	0.589640	0.057*	
C6	−0.0831 (2)	0.2295 (2)	0.74625 (15)	0.0451 (4)	
C7	0.1429 (2)	0.3944 (2)	0.94683 (13)	0.0388 (3)	
C8	0.1195 (3)	0.2573 (2)	0.98958 (17)	0.0524 (4)	
H8	0.029765	0.145119	0.945410	0.063*	
C9	0.2324 (3)	0.2922 (3)	1.09848 (19)	0.0610 (5)	
H9	0.217277	0.201394	1.127299	0.073*	
C10	0.3670 (3)	0.4566 (2)	1.16652 (17)	0.0519 (4)	
H10	0.441229	0.476596	1.239984	0.062*	
C11	0.3892 (2)	0.5915 (2)	1.12302 (14)	0.0414 (4)	
C12	0.2776 (2)	0.5599 (2)	1.01376 (14)	0.0397 (3)	
H12	0.293260	0.650900	0.985061	0.048*	
C13	0.6424 (3)	0.7978 (3)	1.29014 (18)	0.0632 (5)	
H13A	0.576735	0.780929	1.349543	0.095*	
H13B	0.731248	0.917131	1.317583	0.095*	
H13C	0.704699	0.720780	1.276837	0.095*	
N4	0.13965 (18)	0.61019 (17)	0.70978 (11)	0.0396 (3)	
N3	0.31604 (17)	0.54040 (16)	0.57426 (11)	0.0368 (3)	
N2	0.2417 (3)	0.8847 (2)	0.85299 (15)	0.0634 (5)	
H2A	0.143870	0.846000	0.876513	0.076*	
H2B	0.320263	0.991812	0.888534	0.076*	
N1	0.5973 (2)	0.74522 (19)	0.58440 (14)	0.0527 (4)	
H1A	0.606000	0.666365	0.526465	0.063*	
H1B	0.684496	0.848902	0.615032	0.063*	
N5	0.03559 (19)	0.37602 (17)	0.83726 (12)	0.0425 (3)	
H5	0.047786	0.472628	0.827087	0.051*	
O1	−0.1275 (2)	0.08145 (17)	0.74876 (13)	0.0675 (4)	
O2	0.51589 (19)	0.76002 (16)	1.18149 (11)	0.0584 (4)	
S1	0.00691 (5)	0.28602 (5)	0.54469 (3)	0.04131 (14)	

### (II) 2-[(4,6-Diaminopyrimidin-2-yl)sulfanyl]-N-(3-methoxyphenyl)acetamide Atomic displacement parameters (Å^2^)

**Table:**

	*U*^11^	*U*^22^	*U*^33^	*U*^12^	*U*^13^	*U*^23^
C1	0.0530 (9)	0.0339 (8)	0.0391 (8)	0.0203 (7)	0.0146 (7)	0.0113 (6)
C2	0.0476 (9)	0.0276 (7)	0.0427 (8)	0.0118 (6)	0.0105 (7)	0.0081 (6)
C3	0.0413 (8)	0.0308 (7)	0.0357 (7)	0.0122 (6)	0.0090 (6)	0.0106 (6)
C4	0.0364 (7)	0.0323 (7)	0.0323 (7)	0.0130 (6)	0.0046 (6)	0.0113 (6)
C5	0.0302 (7)	0.0491 (10)	0.0466 (9)	0.0049 (7)	0.0058 (6)	0.0087 (7)
C6	0.0359 (8)	0.0407 (9)	0.0453 (9)	0.0031 (7)	0.0145 (7)	0.0103 (7)
C7	0.0421 (8)	0.0363 (8)	0.0376 (8)	0.0151 (6)	0.0163 (6)	0.0115 (6)
C8	0.0598 (10)	0.0365 (9)	0.0551 (10)	0.0112 (8)	0.0152 (8)	0.0187 (8)
C9	0.0724 (13)	0.0485 (10)	0.0673 (12)	0.0203 (9)	0.0154 (10)	0.0348 (9)
C10	0.0593 (10)	0.0522 (10)	0.0500 (10)	0.0255 (9)	0.0104 (8)	0.0248 (8)
C11	0.0459 (8)	0.0374 (8)	0.0417 (8)	0.0201 (7)	0.0111 (7)	0.0119 (7)
C12	0.0472 (8)	0.0330 (8)	0.0397 (8)	0.0163 (7)	0.0127 (7)	0.0139 (6)
C13	0.0604 (11)	0.0612 (12)	0.0535 (11)	0.0200 (10)	−0.0085 (9)	0.0171 (9)
N4	0.0433 (7)	0.0355 (7)	0.0396 (7)	0.0161 (6)	0.0133 (5)	0.0117 (5)
N3	0.0382 (6)	0.0315 (6)	0.0349 (6)	0.0108 (5)	0.0090 (5)	0.0082 (5)
N2	0.0777 (11)	0.0396 (8)	0.0652 (10)	0.0191 (8)	0.0375 (9)	0.0052 (7)
N1	0.0504 (8)	0.0339 (7)	0.0568 (9)	0.0046 (6)	0.0241 (7)	0.0037 (6)
N5	0.0473 (7)	0.0336 (7)	0.0391 (7)	0.0098 (6)	0.0117 (6)	0.0108 (5)
O1	0.0721 (9)	0.0383 (7)	0.0593 (8)	−0.0068 (6)	0.0068 (7)	0.0129 (6)
O2	0.0670 (8)	0.0390 (7)	0.0513 (7)	0.0153 (6)	−0.0123 (6)	0.0118 (5)
S1	0.0384 (2)	0.0372 (2)	0.0341 (2)	0.00654 (16)	0.00462 (15)	0.00617 (16)

### (II) 2-[(4,6-Diaminopyrimidin-2-yl)sulfanyl]-N-(3-methoxyphenyl)acetamide Geometric parameters (Å, º)

**Table:**

C1—N4	1.358 (2)	C8—C9	1.375 (3)
C1—C2	1.374 (2)	C8—H8	0.9300
C1—N2	1.361 (2)	C9—C10	1.378 (3)
C2—C3	1.393 (2)	C9—H9	0.9300
C2—H2	0.9300	C10—C11	1.387 (3)
C3—N1	1.339 (2)	C10—H10	0.9300
C3—N3	1.357 (2)	C11—O2	1.363 (2)
C4—N4	1.325 (2)	C11—C12	1.383 (2)
C4—N3	1.327 (2)	C12—H12	0.9300
C4—S1	1.7721 (17)	C13—O2	1.418 (2)
C5—C6	1.510 (3)	C13—H13A	0.9600
C5—S1	1.8126 (18)	C13—H13B	0.9600
C5—H5A	0.9700	C13—H13C	0.9600
C5—H5B	0.9700	N2—H2A	0.8600
C6—O1	1.224 (2)	N2—H2B	0.8600
C6—N5	1.345 (2)	N1—H1A	0.8600
C7—C12	1.381 (2)	N1—H1B	0.8600
C7—C8	1.397 (2)	N5—H5	0.8600
C7—N5	1.409 (2)		
			
N4—C1—C2	122.38 (14)	C8—C9—H9	118.7
N4—C1—N2	115.28 (15)	C9—C10—C11	118.53 (17)
C2—C1—N2	122.31 (16)	C9—C10—H10	120.7
C1—C2—C3	117.36 (14)	C11—C10—H10	120.7
C1—C2—H2	121.3	O2—C11—C12	115.38 (14)
C3—C2—H2	121.3	O2—C11—C10	124.50 (15)
N1—C3—N3	116.77 (14)	C12—C11—C10	120.12 (16)
N1—C3—C2	121.86 (14)	C7—C12—C11	120.61 (15)
N3—C3—C2	121.37 (14)	C7—C12—H12	119.7
N4—C4—N3	128.70 (14)	C11—C12—H12	119.7
N4—C4—S1	119.42 (12)	O2—C13—H13A	109.5
N3—C4—S1	111.88 (11)	O2—C13—H13B	109.5
C6—C5—S1	111.79 (12)	H13A—C13—H13B	109.5
C6—C5—H5A	109.3	O2—C13—H13C	109.5
S1—C5—H5A	109.3	H13A—C13—H13C	109.5
C6—C5—H5B	109.3	H13B—C13—H13C	109.5
S1—C5—H5B	109.3	C4—N4—C1	114.83 (14)
H5A—C5—H5B	107.9	C4—N3—C3	115.33 (13)
O1—C6—N5	124.17 (17)	C1—N2—H2A	120.0
O1—C6—C5	122.10 (15)	C1—N2—H2B	120.0
N5—C6—C5	113.70 (15)	H2A—N2—H2B	120.0
C12—C7—C8	119.78 (15)	C3—N1—H1A	120.0
C12—C7—N5	116.25 (14)	C3—N1—H1B	120.0
C8—C7—N5	123.97 (15)	H1A—N1—H1B	120.0
C9—C8—C7	118.46 (17)	C6—N5—C7	129.44 (15)
C9—C8—H8	120.8	C6—N5—H5	115.3
C7—C8—H8	120.8	C7—N5—H5	115.3
C10—C9—C8	122.51 (17)	C11—O2—C13	117.88 (14)
C10—C9—H9	118.7	C4—S1—C5	103.05 (8)
			
N4—C1—C2—C3	−0.1 (2)	S1—C4—N4—C1	−178.68 (11)
N2—C1—C2—C3	177.81 (16)	C2—C1—N4—C4	−1.3 (2)
C1—C2—C3—N1	−178.60 (15)	N2—C1—N4—C4	−179.36 (14)
C1—C2—C3—N3	1.3 (2)	N4—C4—N3—C3	−0.6 (2)
S1—C5—C6—O1	−95.98 (18)	S1—C4—N3—C3	179.78 (10)
S1—C5—C6—N5	82.23 (16)	N1—C3—N3—C4	178.93 (14)
C12—C7—C8—C9	0.2 (3)	C2—C3—N3—C4	−1.0 (2)
N5—C7—C8—C9	−179.56 (17)	O1—C6—N5—C7	5.5 (3)
C7—C8—C9—C10	0.0 (3)	C5—C6—N5—C7	−172.71 (14)
C8—C9—C10—C11	−0.1 (3)	C12—C7—N5—C6	168.50 (15)
C9—C10—C11—O2	179.53 (18)	C8—C7—N5—C6	−11.7 (3)
C9—C10—C11—C12	0.1 (3)	C12—C11—O2—C13	−175.89 (16)
C8—C7—C12—C11	−0.2 (2)	C10—C11—O2—C13	4.7 (3)
N5—C7—C12—C11	179.57 (14)	N4—C4—S1—C5	−8.27 (14)
O2—C11—C12—C7	−179.39 (14)	N3—C4—S1—C5	171.36 (11)
C10—C11—C12—C7	0.1 (2)	C6—C5—S1—C4	−80.14 (14)
N3—C4—N4—C1	1.8 (2)		

### (II) 2-[(4,6-Diaminopyrimidin-2-yl)sulfanyl]-N-(3-methoxyphenyl)acetamide Hydrogen-bond geometry (Å, º)

**Table:**

*D*—H···*A*	*D*—H	H···*A*	*D*···*A*	*D*—H···*A*
N5—H5···N4	0.86	2.15	2.861 (3)	140
C8—H8···O1	0.93	2.34	2.911 (3)	120
N1—H1*A*···N3^i^	0.86	2.21	3.035 (3)	162
N1—H1*B*···O1^ii^	0.86	2.08	2.891 (3)	157
N2—H2*B*···O2^iii^	0.86	2.55	3.210 (3)	135
C2—H2···O2^iii^	0.93	2.59	3.272 (3)	130

Symmetry codes: (i) −*x*+1, −*y*+1, −*z*+1; (ii) *x*+1, *y*+1, *z*; (iii) −*x*+1, −*y*+2, −*z*+2.
